# Live Attenuated Rev-Independent Nef¯SIV Enhances Acquisition of Heterologous SIVsmE660 in Acutely Vaccinated Rhesus Macaques

**DOI:** 10.1371/journal.pone.0075556

**Published:** 2013-09-30

**Authors:** Siddappa N. Byrareddy, Mila Ayash-Rashkovsky, Victor G. Kramer, Sandra J. Lee, Mick Correll, Francis J. Novembre, Francois Villinger, Welkin E. Johnson, Agneta von Gegerfelt, Barbara K. Felber, Ruth M. Ruprecht

**Affiliations:** 1 Dana-Farber Cancer Institute, Boston, Massachusetts, United States of America; 2 Harvard Medical School, Boston, Massachusetts, United States of America; 3 Department of Biostatistics and Computational Biology, Dana-Farber Cancer Institute, Boston, Massachusetts, United States of America; 4 Center for Cancer Computational Biology, Dana-Farber Cancer Institute, Boston, Massachusetts, United States of America; 5 Yerkes National Primate Research Center, Emory University, Atlanta, Georgia, United States of America; 6 Department of Microbiology and Immunology, Emory University, Atlanta, Georgia, United States of America; 7 Department of Pathology and Laboratory Medicine, Emory Vaccine Center, Emory University, Atlanta, Georgia, United States of America; 8 Biology Department, Boston College, Boston, Massachusetts, United States of America; 9 Human Retrovirus Pathogenesis Section, Vaccine Branch, Center for Cancer Research, Frederick, Maryland, United States of America; Department of Pathology & Laboratory Medicine, Emory Vaccine Center, Emory University School of Medicine, Atlanta, United States of America.; University of Pittsburgh Center for Vaccine Research, United States of America

## Abstract

**Background:**

Rhesus macaques (RMs) inoculated with live-attenuated Rev-Independent Nef¯ simian immunodeficiency virus (Rev-Ind Nef¯SIV) as adults or neonates controlled viremia to undetectable levels and showed no signs of immunodeficiency over 6-8 years of follow-up. We tested the capacity of this live-attenuated virus to protect RMs against pathogenic, heterologous SIVsmE660 challenges.

**Methodology/Principal Findings:**

Three groups of four RM were inoculated with Rev-Ind Nef¯SIV and compared. Group 1 was inoculated 8 years prior and again 15 months before low dose intrarectal challenges with SIVsmE660. Group 2 animals were inoculated with Rev-Ind Nef¯SIV at 15 months and Group 3 at 2 weeks prior to the SIVsmE660 challenges, respectively. Group 4 served as unvaccinated controls. All RMs underwent repeated weekly low-dose intrarectal challenges with SIVsmE660. Surprisingly, all RMs with acute live-attenuated virus infection (Group 3) became superinfected with the challenge virus, in contrast to the two other vaccine groups (Groups 1 and 2) (*P*=0.006 for each) and controls (Group 4) (*P*=0.022). Gene expression analysis showed significant upregulation of innate immune response-related chemokines and their receptors, most notably CCR5 in Group 3 animals during acute infection with Rev-Ind Nef¯SIV.

**Conclusions/Significance:**

We conclude that although Rev-Ind Nef¯SIV remained apathogenic, acute replication of the vaccine strain was not protective but associated with increased acquisition of heterologous mucosal SIVsmE660 challenges.

## Introduction

According to the recent UNAIDS estimates [www.unaids.org], approximately 40 million people are infected with HIV worldwide. The only cost-effective strategy to curb this epidemic will be an effective vaccine [1]. Among the various strategies tested thus far, live-attenuated simian immunodeficiency virus (LASIV) vaccination was tested in primate models and shown to provide good protection against homologous virus challenges [2-6] but less so against heterologous virus challenges [7-11]. Parental SIVmac239 was attenuated using various approaches, including deletion of nonessential genes, mutations in virus sequences, or swapping of important gene functions. The most popular LASIV studied in the rhesus macaque (RM) model is SIVmac239, that lacks a functional *nef* gene (SIVΔnef). Animals infected with SIVΔnef maintained persistently low viral loads with stable CD4 counts over several years and were able to resist challenges with high-dose of homologous *nef*+ SIV [2]. Further virus attenuations [12-18], as in the SIVmac239Δ3 isolate [16], decreased the replicative capacity further, but also reduced levels of protection; when SIVmac239Δ3 was used to vaccinate neonatal RMs, these animals were unable to control viremia and showed signs of disease progression, including development of AIDS [14]. Together, these findings led to the threshold hypothesis [5,19]. Even RMs given SIVmac239Δ3 as adults developed signs of immune dysregulation over time: more than half showed T-cell depletion after 6-8 years of follow-up and 18% developed AIDS [14,20]. These data indicated that deletion of *nef* in the SIVmac239 genome did not fully abrogate pathogenicity but markedly delayed disease progression.

Interestingly, pathogenicity of *nef-*deleted HIV has also been reported in a unique cohort of long-term non-progressors (LTNP) of the Sydney Blood Bank, where people were infected with *nef*-deleted HIV via transfusion from a single donor. These recipients were initially healthy but some of them exhibited a slow increase in viral loads, which ultimately led to disease progression and opportunistic infections [21,22].

We have reported a non-pathogenic LASIVmac239 derivative, in which Rev and the Rev-Responsive Element (RRE) were replaced by the constitutive transport element (CTE) of simian retrovirus type D (SIV-D). The resulting Rev-Independent *nef*-deleted SIV, termed Rev-Ind Nef¯SIV, is critically dependent on CTE for viral RNA (vRNA) export into the cytoplasm for its replication, although the transplanted SIV-D CTE significantly slowed the export and resulted in lower viral replication in vitro and in vivo [23-26]. RMs inoculated with Rev-Ind Nef¯SIV showed low viral loads during acute infection and exhibited long-lasting, persistent humoral and cellular SIV-specific immune responses [26,27]. In addition, RMs infected with Rev-Ind Nef¯SIV as adults or neonates and followed prospectively for 6-8 years controlled this virus and exhibited no signs of immune dysfunction or progression to AIDS [25-27]. Thus, Rev-Ind Nef¯SIV not only appeared to represent a live-attenuated SIV strain but may also be truly non-pathogenic since not even RMs infected as neonates showed signs of SIV disease. Given this safety profile, we sought to test the efficacy of Rev-Ind Nef¯SIV as a vaccine candidate against challenge with a heterologous virus.

Several studies examined the need for LASIV-induced protective responses to develop over time, with some studies describing time-dependent elicitation of protective responses by the attenuated virus [6,9,10,28-30] and (reviewed in 3,5). However, neither very short nor very long time spans - >8 years – between the initial vaccination and pathogenic virus challenge have been investigated. Here, we tested the effect of different time intervals between infection with live-attenuated Rev-Ind Nef¯SIV and intrarectal (i.r.) repeat low-dose challenges with heterologous SIVsmE660.

## Results

### Immunization of RMs with Rev-Ind Nef¯SIV and immune responses

**Figure 1:**
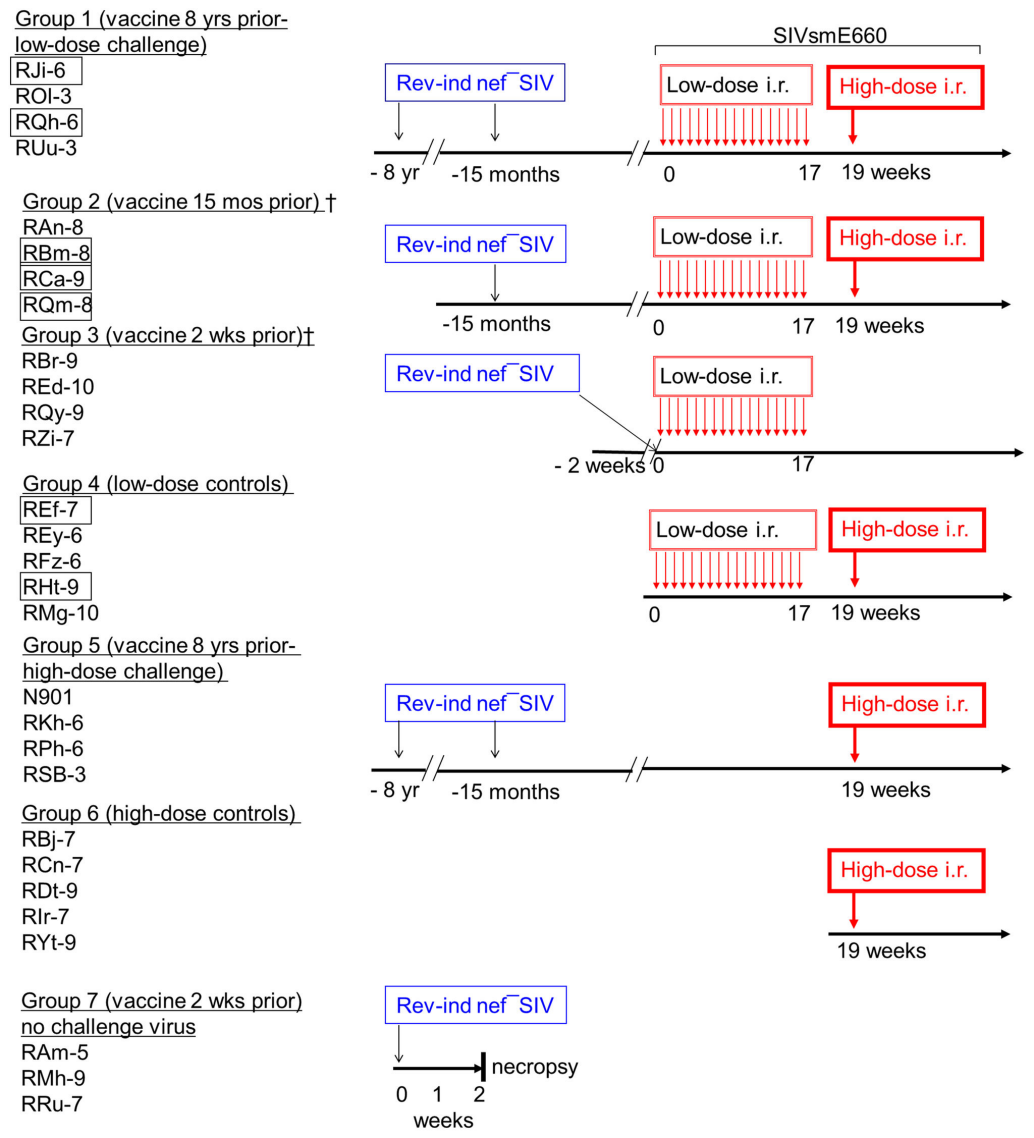
Vaccination strategy with Rev-Ind Nef¯SIV and SIVsmE660 challenges. RMs in Groups 1(low-dose challenge group) 5 and (high-dose only challenge group) had been vaccinated with Rev-Ind Nef¯SIV >8 yrs ago and boosted along with Group 2 monkeys; RMs in Group 2 were vaccinated with Rev-Ind Nef¯SIV 15 months prior to SIVsmE660 low-dose challenges, RMs in Group 3 were vaccinated with Rev-Ind Nef¯SIV 2 weeks prior to SIVsmE660 low-dose challenges; Group 4 consisted of naïve RMs that served as controls for SIVsmE660 challenges; Groups 6, served as control unvaccinated group was challenged with a high-dose of SIVsmE660 only and Group 7 received only Rev-Ind Nef¯SIV and was followed for 2 weeks. Groups 1-4 received the maximum of 17 weekly low-dose i.r. challenges with SIVsmE660 (300 TCID_50_ as measured by TZM-bl assay). RMs that did not become systemically infected after the 17 low-dose challenges (boxed names) were rechallenged i.r. with a single high dose of SIVsmE660 (990 TCID_50_) and became infected (rechallenged Group 1-2 and high-dose control Group 6). Blue indicates the live attenuated vaccine strain (Rev-Ind Nef¯SIV). A fifth monkey (RMw-6) did not fulfill the criteria of “vaccine take” (transient low-dose Rev-Ind Nef¯SIV infection with vRNA of 1080 copies/ml at 2 weeks post-vaccination, the expected time of peak vaccine virus viremia); therefore, data from RWm-6 were not included in the analysis. Thin red arrows stand for the weekly low-dose i.r. exposures to the challenge virus, heterologous SIVsmE660; the thick red arrows represent single high-dose i.r. challenges with SIVsmE660.

A total of 8 RMs were infected as neonates (4 RMs) or adults (4 RMs) with the live attenuated, Rev-Ind Nef¯SIV and monitored prospectively for approximately 8 years (Groups 1 and 5, Figure 1). After the initial acute viremia phase, plasma vRNA loads were persistently below the threshold of the assay [25,26]. No signs of immune dysfunction or AIDS were observed, and all animals showed persistent humoral and cellular SIV-specific immune responses, consistent with chronic infection [25,26]. Multicolor flow cytometric analysis demonstrated preservation of the central memory (CM) subset of T cells [25,26]. In efforts to boost immune responses, we re-inoculated these monkeys with a high-dose (1.6 x 10^4^ TCID_50_) of Rev-Ind Nef¯SIV i.v. at 15 months prior to the pathogenic SIVsmE660 challenges (-15 mos). In parallel, we challenged 4 naïve control monkeys to ensure the infectivity of the vaccine virus inoculum (Group 2 “vaccine 15 mos prior”). The latter 4 monkeys had peak vRNA loads of 10^4^-10^5^ copies/ml at week 2 post-inoculation (p.i.), but viral loads declined rapidly thereafter and became undetectable. In contrast, none of the vaccinees in Group 1 or 5 had measurable viremia upon re-inoculation. All vaccinees in Group 2 seroconverted by week 9 post-inoculation as measured by Western blot analysis; all vaccinees in Group 1 and 5 remained seropositive through 8 years of follow-up. Cellular SIV-specific immune responses were detected in both RM groups as measured by ELISPOT assay for interferon (IFN)-γ-producing cells after stimulation with peptide pools for SIV Gag [27]. Hematological values, including absolute numbers and percentage of CD3^+^CD4^+^cells, CD4^+^/CD8^+^ ratios, the percent CD4^+^CD29^+^ memory T cells, and the number of platelets remained stable in Group 1 and 5 vaccinees during the post-boost period as well as in Group 2 RMs (data not shown). No differences were observed in the frequencies of CM populations of CD4^+^ cells (CD28^+^CD95^+^) and CD8^+^ cells (CD45RA^-^CCR7^+^) as measured by multicolor flow cytometric analysis (data not shown).

### Repeated low-dose and high dose SIVsmE660 i.r. challenges

As it is unlikely that any AIDS vaccine recipient will be exposed to an HIV variant exactly matching the immunogens, vaccine efficacy needs to be evaluated using heterologous challenge viruses [31]. Accordingly, we challenged our cohort of Rev-Ind Nef¯SIV-vaccinated RMs with multiple low-dose mucosal exposures of SIVsmE660 as outlined in Figure 1. The overall sequence difference between these two strains ranges from 8-26% at the amino acid level. The highest difference is in Tat (26%), followed by Env (15.1%), Vpr (12%), Pol (8.3%), and Gag (8%) [10]. The pathogenic SIVsmE660 exposure of the different vaccine groups occurred at different time intervals after the initial vaccination and ranged from 8 years to 2 weeks.

Viral RNA (vRNA) load determination was complicated by a need to differentiate the challenge virus (SIVsmE660) from the live attenuated vaccine strain (Rev-Ind Nef¯SIV). Since the latter was based upon the molecular clone of SIVmac239, we developed a set of primers that only recognized Rev-Ind Nef¯SIV but not the challenge virus SIVsmE660 [32]. A second assay based upon primers developed by Cline et al. [33], was then used to measure total vRNA. The difference between the two assays reflects vRNA loads due to SIVsmE660 superinfection.

**Figure 2:**
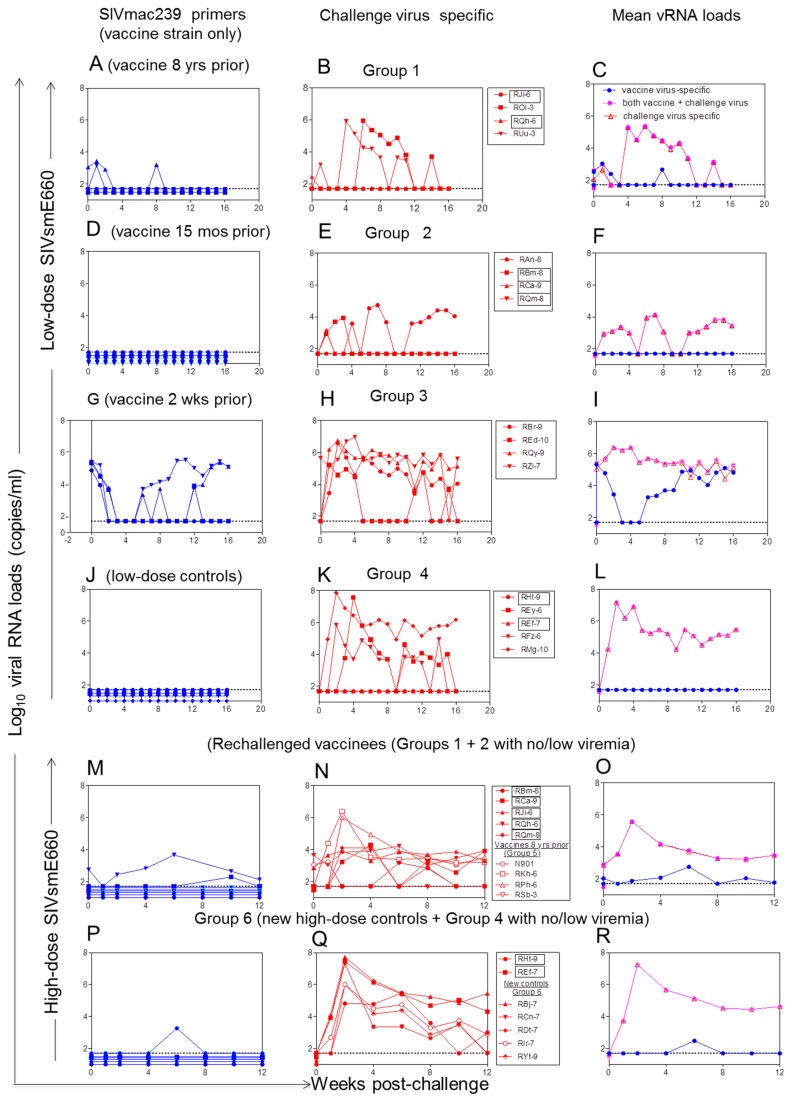
Plasma vRNA loads after low-dose (A-B; D-E; G-H; J-K) or high-dose (M-N; P-Q) SIVsmE660 challenges. Horizontal dashed lines in all panels represent the limit of detection of the RT-PCR assay (50 vRNA copies/ml) [32,33]. RMs in boxes were entered into the single high-dose challenge with SIVsmE660 (thick arrow) shown in M-N and P-Q. The panels in (C, F, I, L, O, R) are mean vRNA loads of each group. Blue, indicates vRNA loads for the live attenuated vaccine strain (Rev-Ind Nef¯SIV), purple indicates vRNA loads both vaccine + challenge virus, and the red line indicates challenge virus-specific vRNA loads.

All vaccinated RMs (Groups 1, 2 and 3) and control Group 4 were challenged up to 17 times i.r. with weekly low-dose SIVsmE660. Animals that remained aviremic after these 17 low doses were given a single high dose of SIVsmE660 (boxed animal names in Figures 1, 2). Remarkably, all 4 RMs of Group 3 challenged with SIVsmE660 2 weeks after receiving the live attenuated Rev-Ind Nef¯SIV (i.e., peak viremia of the vaccine strain, Figure 2G, week 0) became superinfected after only 1 to 3 low-dose SIVsmE660 challenges (Figure 2H). In contrast, some vaccinees of Groups 1 and 2 (Figure 2A-F) as well as control Group 4 remained aviremic (Figure 2J-L). Statistical analysis of this low-dose challenge phase revealed the following for the time-to-first viremia (defined as vRNA >10^4^ copies/ml): Group 3 vs. Groups 1 or 2 (*P*=0.006 for each comparison by Log rank test, two-sided); Group 3 vs. the combined Groups 1 + 2 (*P*<0.001). During the low-dose challenge phase, viremia developed in 3 out of 5 Group 4 controls (Figure 2J-L). Time-to-first-viremia was significantly shorter for Group 3 vaccinees compared to Group 4 controls (*P*=0.022). Clearly, Group 3 vaccinees with acute vaccine strain infection had the highest SIVsmE660 acquisition rate with the fastest time to viremia compared to all other groups.

The fact that not all naïve controls became viremic prompted the high-dose SIVsmE660 rechallenge, after which all virus-only controls (Group 4 RMs with boxed names and a new control Group 6 of naïve monkeys, Figure 1) became viremic (Figure 2P-R). In addition, the 2 RMs from Group 1 and 3 RMs from Group 2 that remained uninfected after the multiple low-dose challenges (Figure 2A-F) as well as Group 5 monkeys inoculated with Rev-Ind Nef¯SIV at -8 years and -15 months (Figure 1) were also subjected to the single high-dose i.r. SIVsmE660 challenge. After the high-dose rechallenge, all RMs became superinfected with the challenge virus except for vaccinee RSb-3 that remained completely protected (Group 5, Figure 2M-N), outlining the limits of protection from acquisition of a heterologous mucosal challenge afforded by our LASIV. In addition, while all animals from Group 1 and 2 except RAn-8 controlled viremia after resolution of the initial SIVsmE660 infection, vaccinees challenged with a single high dose of SIVsmE660 exhibited persistent viremia, albeit at lower set points than Group 5 control animals (Figure 2). Peak vRNA loads due to SIVsmE660 superinfection were also affected by immunization with Rev-Ind Nef¯SIV. Control RMs of Groups 4 and 5 had peak vRNA loads of 10^6^-10^8^ copies/ml. Peak vRNA loads for SIVsmE660 did not differ between Groups 3 and 4. Group 1 vaccinees had peak vRNA loads ranging from 10^4^-10^5^ copies/ml, except in vaccinee RJi-6 (Group 1, Figure 2N), where several blips of below ~10^3^ copies/ml were noted. A significant difference in challenge virus peak vRNA loads was noted for the comparison of vaccinees exposed to the pathogenic SIVsmE660 during the chronic phase of live-attenuated Rev-Ind Nef¯SIV infection (Groups 1 + 2) with that of RMs during acute Rev-Ind Nef¯SIV infection (Group 3) (*P*=0.016 by Wilcoxon rank-sum test, two-sided; Figure 2B and E, versus H).

All newly infected animals and controls seroconverted by week 9 post-challenge as measured by Western blot analysis. We conclude that exposure to pathogenic challenge virus at week 2, the time of peak vaccine strain viremia, not only failed to protect but may even have increased host susceptibility to the heterologous pathogenic virus challenge in comparison with controls. Furthermore, exposure to pathogenic challenge during acute vaccine virus infection also led to significantly higher viral set points and continued replication of both challenge and vaccine strains compared to vaccinees given the live-attenuated virus 15 months to 8 years earlier.

### SIV-specific immune responses before and after low-dose challenges

**Figure 3:**
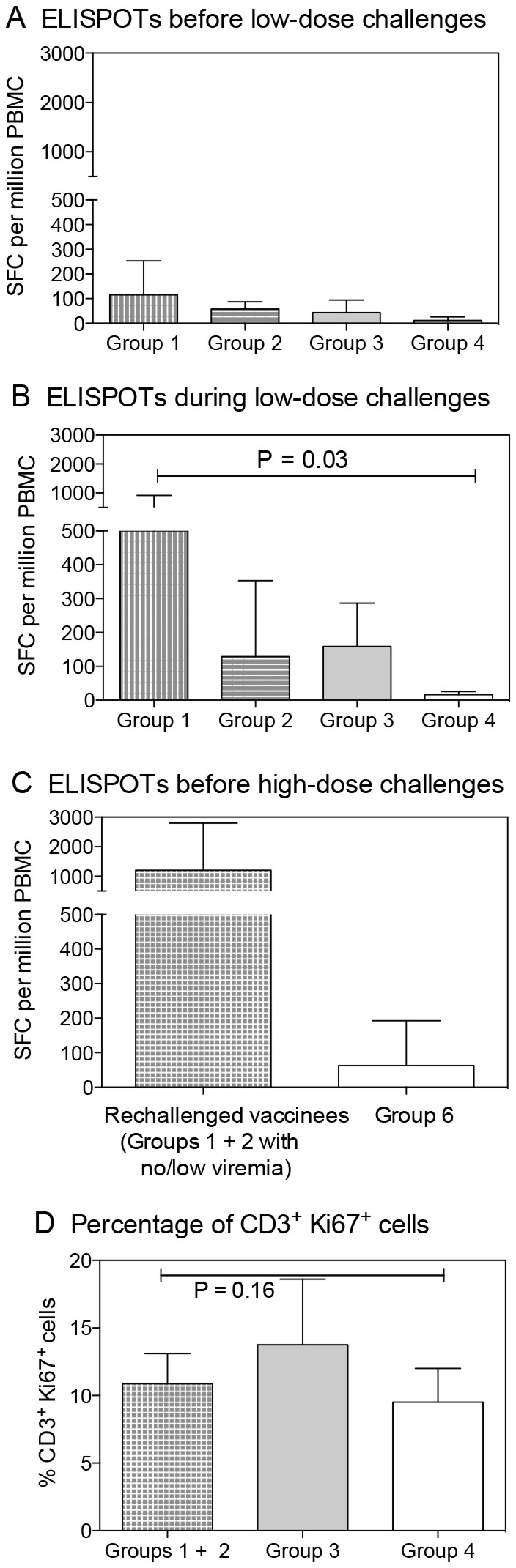
Cellular responses and T cell activation. SIV Gag-specific, IFN-γ-secreting cells were measured by ELISPOT assay (A) before the low-dose SIVsmE660 challenge phase, (B) during the low-dose challenge phase, or (C) after the low-dose phase but before the high dose rechallenge with SIVsmE660 and (D) T cell activation was measured by percentage of CD3^+^ Ki67^+^ cells.

Next, we measured anti-SIV Gag IFN-γ ELISPOT responses before and during the low-dose SIVsmE660 challenges (Figure 3). Only low-level IFN-γ ELISPOT responses were observed among Groups 1, 2, and 3 prior to the repeated low-dose challenges. These responses did not differ significantly among the vaccine groups (*P*=0.19; Figure 3A). However, during the low-dose challenges, SIV Gag-directed IFN-γ ELISPOT responses increased among all three vaccine recipient groups and differed from controls (*P*=0.03; Figure 3B), particularly in Group 1, even though the difference between the vaccine groups never reached significant statistical difference. SIV Gag-specific IFN-γ ELISPOT responses on the day of the single high-dose SIVsmE660 rechallenge seen among the vaccinees of Groups 1 and 2 that had not developed persistent challenge virus viremia during the low-dose SIVsmE660 challenge phase (boxed RMs in Figures 1 and 2B,E) had increased above the levels seen prior to the low-dose challenge virus exposures, suggesting some boosting by the repeat exposure (Figure 3C). Of interest was the finding that SIVgag specific responses did not correlate with protection despite the equivalent levels of such responses between animals from Groups 2 and 3 in particular.

Given the higher acquisition rate observed in Group 3 animals that were exposed to the pathogenic challenge virus at the time of peak vaccine virus viremia, we sought to test PBMC for evidence of T-cell activation by staining for Ki67, CD69, HLA-DR, and CCR5 by multicolor flow cytometry. Although staining for Ki67 on CD3+ cells was higher for Group 3 animals compared to those of Groups 1+2 and 4, statistical significance was not reached (*P*=0.16) (Figure 3D). Furthermore, in consideration of the potential inflammation induced by Rev-Ind Nef¯SIV in Group 3 monkeys, we also measured the relative representation of total CD4^+^ T cells, CD4^+^CD45RA^-^CCR5^+^, CD45RA^-^CCR7^+^ memory and CD4^+^ CD28^+^ CD95^+^ central memory cells before and 2 weeks after post-infection (Group 7). As shown in Figure S1, higher percentages of these populations were noted at 2 weeks post Rev-Ind Nef¯SIV infection relative to baseline confirming the microarray data. However, phenotypic analysis did not reach statistical significance, likely due to the small number of animals.

### The potential influence of host restriction factors on viral loads

The animals were enrolled and grouped years before the influence of MHC class I or TRIM5α alleles on viral loads became known. However, it is now generally recognized that low-dose SIVsmE660 challenges may be influenced by TRIM5α alleles [7,34]. We therefore typed all vaccinees and controls enrolled in this study for their MHC Class I and TRIM5α genotypes (Table 1; RMs in Group 6 are not included in this discussion since they did not undergo SIVsmE660 challenge). While the distribution of TRIM5a alleles was not perfectly equal among groups, it remains comparable with only 1 sensitive genotype in Groups 2, 3 and 5 while the other groups comprised a blend of moderately to highly restrictive genotypes. Of note, neither TRIM5α nor the MHC B*008 or B*017 appeared to greatly affect the susceptibility of infection within any defined group except perhaps for Group 2 (Table 1). Of note though, the low-dose control Group 5 comprised four out of five “restrictive” genotypes, which may have lead to two RMs requiring 17 weekly low-dose SIVsmE660 challenges. However, the other 2 animals possessing a restrictive genotype as well as the fifth, with a moderate genotype, were infected rapidly during the low dose challenge series, suggesting that perhaps other yet to be defined factors played a role. Of note, all controls, regardless of their TRIM5α or MHC genotype, became infected after the single high-dose SIVsmE660 challenge. Finally, we also performed statistical analysis by combining restrictive, moderate, susceptibility to infection of TRIM5α alleles of all the vaccinees vs. control RMs for virus acquisition. We noted that vaccinees and control RMs experienced comparable acquisition rates over the first 4 challenges and after that, vaccinees showed slower acquisition compared to controls (P=0.045; based on Mantel-Cox test) which is in agreement with recently published studies [35,36], suggesting that TRIM5α played a minor role at best in acquisition of infection in this study.

**Table 1:** TRIM5α and MHC Genotypes.

RM #	TRIM5α genotype	Susceptibility to infection	MHC genotypes			# SIVsmE660 challenges	
			A01	B08	B17	Low-dose High-dose	
Group 1 (vaccine 8 yrs prior, followed by low-dose repeated challenge)							
RJi-6	TFP/ Q	moderate	–	–	–	17	1
ROI-3	TFP/ TFP	restrictive	–	–	–	5	0
RQh-6	Q/ CYPA	moderate	–	–	–	17	1
RUu-3	TFP/ TFP	restrictive	–	+	–	4	0
Group 2 (vaccine 15 mos prior, followed by low-dose repeated challenges)							
RAn-8	Q/ Q	high	–	–	–	6	0
RBm-8	TFP/ Q	moderate	+	–	–	17	1
RCa-9	Q/ CYPA	moderate	–	–	–	17	1
RQm8	Q/CYPA	moderate	+	–	–	17	1
Group 3 (vaccine 2 wks prior, followed by low-dose repeated challenges)							
RBr-9	Q/CYPA	moderate	+	–	+	1	0
REd-10	TFP/ TFP	restrictive	+	–	–	1	0
RQy-9	Q/ CYPA	moderate	–	–	–	1	0
RZi-7	Q/TRIM Q	high	–	–	–	1	0
Group 4 (low-dose controls for low dose repeated challenges)							
REf-7	TFP/ TFP	restrictive	+	–	–	17	1
REy-6	TFP/ TFP	restrictive	–	–	–	4	0
RFz-6	TFP/ TFP	restrictive	–	–	–	2	0
RHt-9	TFP/ TFP	restrictive	+	–	–	17	1
RMg-10	Q/CYPA	moderate	+	–	–	1	0
Group 5 (vaccine 8 yrs prior, followed by high-dose challenge)							
N901	TFP/ TFP	restrictive	–	–	–	NA	1
RKh-6	TFP/ Q	moderate	–	–	–	NA	1
RPh-6	TFP/ TFP	restrictive	–	–	–	NA	1
RSB3	TFP/ CYPA	moderate	–	–	–	NA	1
Group 6 (high-dose controls for high dose challenge)							
RBj-7	Q/ Q	high	–	–	–	NA	1
RCn-7	TFP/TFP	restrictive	–	–	+	NA	1
RDt-9	TFP/ TFP	restrictive	–	–	–	NA	1
RIr-7	TFP/ CYPA	moderate	–	–	–	NA	1
RYt-9	TFP/ TFP	restrictive	–	+	–	NA	1
Group 7 (vaccine only for 2 wks)							
RAm-5	TFP/ TFP	restrictive	+	–	+	no	no
RMh-9	TFP/ Q	moderate	+	–	–	no	no
RRu-7	TFP/ Q	moderate	–	+	–	no	no

### Gene expression analysis of RMs 2 weeks after i.v. vaccination with Rev-Ind Nef¯SIV (Group 7)

**Figure 4:**
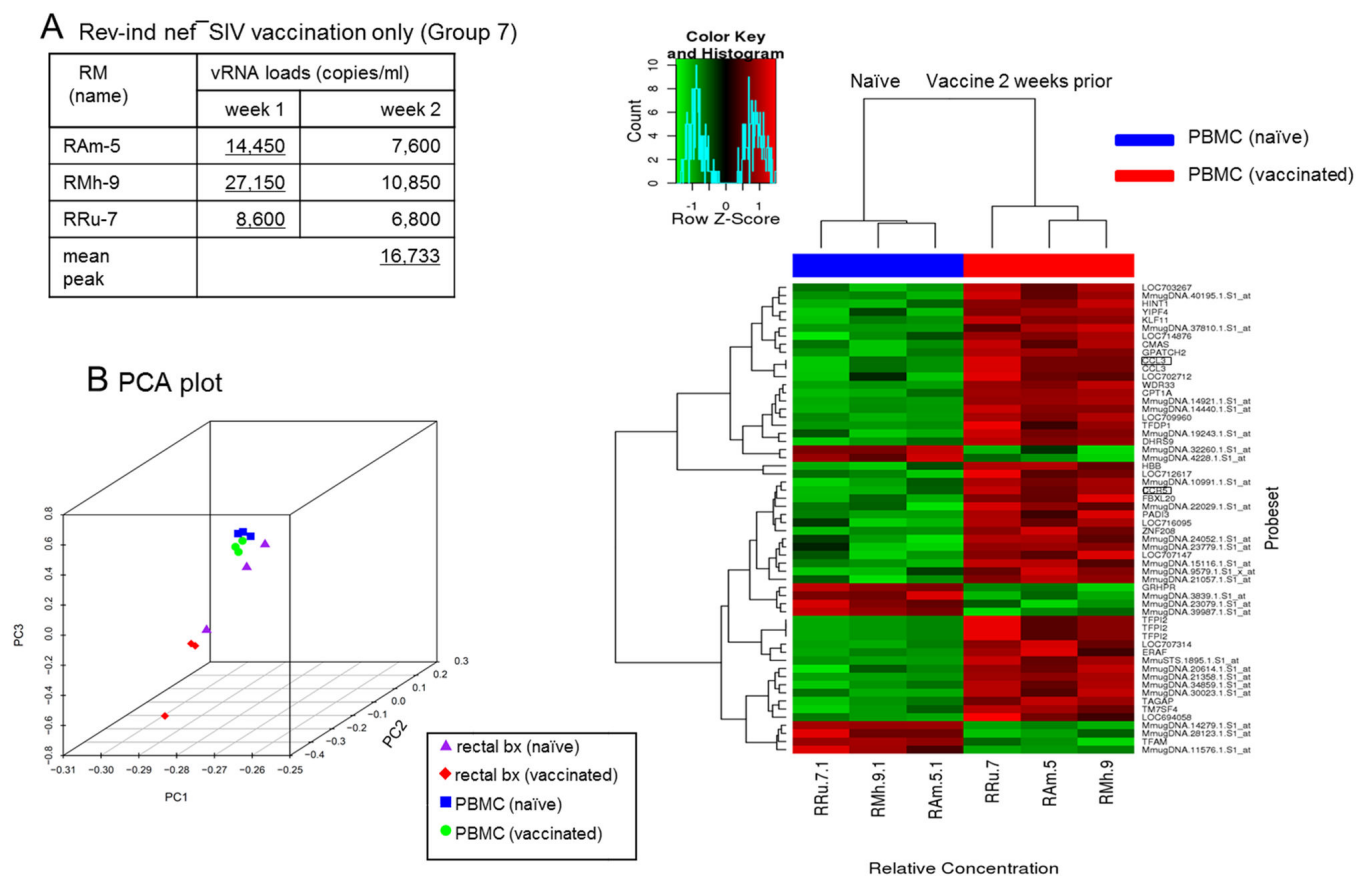
vRNA load measurements, principal component analysis (PCA) and heatmap analysis. (A) vRNA loads were measured at 2 weeks after Rev-Ind Nef¯SIV vaccination (Group 7; 3 RMs) The RMs were subsequently euthanized to collect rectal tissues and PBMC. (B) PCA was performed on log_2_ expression measures for autologous PBMC and rectal biopsy specimens collected before and two weeks after inoculation with the live attenuated vaccine strain, Rev-Ind Nef¯SIV. Infected and naïve PBMC samples were centered and grouped together, indicating that the samples were nearly homogenous. In contrast, we observed heterogeneity in rectal tissues. (C) Hierarchical clustering of differentially expressed genes in PBMC. Supervised analysis identified approximately 100 differentially expressed genes between PBMC of the RMs collected before and after vaccination using a significance threshold of a false discovery rate) <5% and log_2_ fold-change >1 was analyzed using average-linkage hierarchical clustering with a Pearson correlation coefficient distance metric. We noted a clear upregulation of expression of a set of chemokines and chemokine receptors, most notably CCL3, and CCL5 (highlighted).

To understand the potential mechanism that led to the increased susceptibility of RMs to challenge virus acquisition 2 weeks after the inoculation of Rev-Ind Nef¯SIV, three naïve RMs were enrolled for PBMC and rectal tissue gene-expression profiling before and after vaccination with Rev-Ind Nef¯SIV (Group 7, Figure 1 and Table 1); these additional RMs were vaccinated similar to Group 3 animals and monitored for plasma vRNA loads (Figure 4A). At week 2, all three Group 7 RMs were euthanized, and PBMC and rectal biopsies were collected using identical procedures pre-vaccination, thus allowing us to compare paired samples from each RM, with pre-vaccination samples as autologous controls for microarray analysis (Methods; Figure 4B). Unsupervised analysis using PCA showed a tight grouping of PBMC samples, while rectal tissues exhibited greater heterogeneity.

Supervised analysis identified approximately 100 individual genes that were differentially expressed in PBMC (Figure 4C) when comparing pre- and post-vaccination samples but none in rectal biopsies. We noted a clear upregulation of expression of a set of chemokine and chemokine receptors, most notably CCL3, also known as macrophage inflammatory protein-1α (MIP-1α), and C-C chemokine receptor 5 (CCR5). We further validated the increased expression of CCR5 by quantitative qPCR (data not shown). Increased expression was seen also for several genes involved in different cellular processes, including cell cycle, signal transduction and chromatin remodeling (Figure 4C). In contrast, gut biopsies exhibited no such differences, suggesting that relatively few changes occurred in the gut during acute infection with Rev-Ind Nef¯SIV or that tissue sample heterogeneity, the relatively few lymphocytes residing in the mucosal tissues, and the low number of RMs available did not permit observation of significant differences.

**Figure 5:**
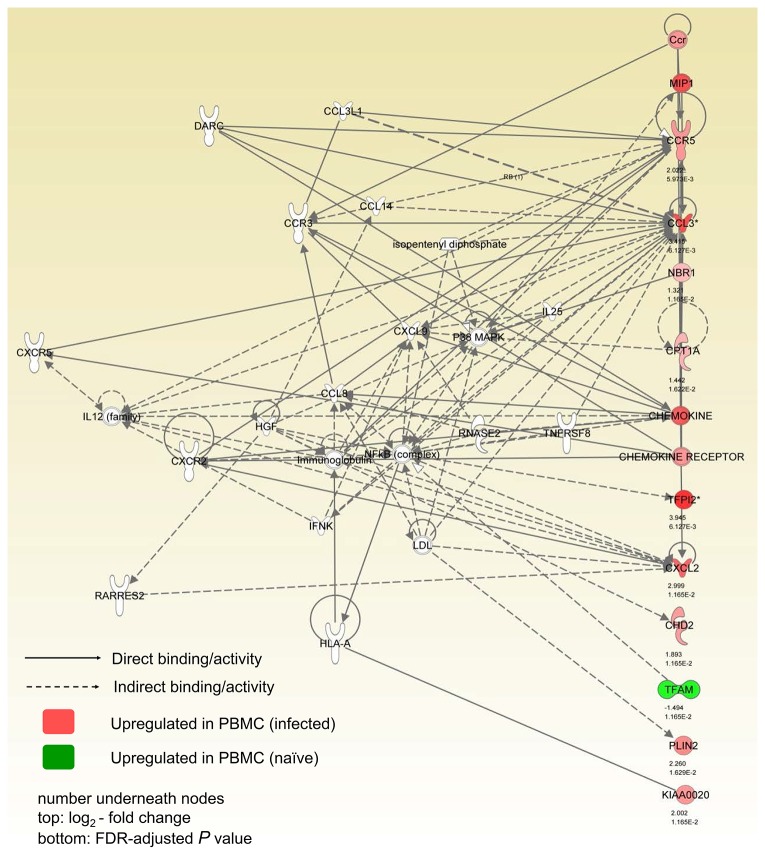
Network pathway analysis. Network pathway analyses were performed using the Ingenuity Pathway Analysis Network tool. Node colors show the average gene expression levels of RMs vaccinated with Rev-Ind Nef¯SIV relative to the average before vaccination (red, relative increase in expression; green, relative decrease in expression). CCR5, CCL3 and other signaling molecules were upregulated and were directly and indirectly involved with other immune system pathways. The numbers underneath the nodes are 1) log_2_-fold change, and 2) FDR-adjusted *P* values.

Evaluation of these results with the Ingenuity Pathway Analysis (IPA) showed that the top network genes regulated by Rev-Ind Nef¯SIV include functional groups responsible for hematopoiesis, cellular movement, and immune cell trafficking. Several functionally linked genes were upregulated as shown in Figure 5. Network analysis revealed that a set of upregulated chemokines and chemokine receptors formed clusters/cores with NF-ĸB and other signaling molecules (Figure 5). Collectively, these data indicate Rev-Ind Nef¯SIV vaccination induced immune activation within 2 weeks of infection that was clearly discernible in PBMC but not in rectal tissues. In turn, immune activation caused by the live attenuated vaccine virus may have increased host susceptibility to the heterologous SIVsmE660 during the low-dose challenges and facilitated its dissemination.

## Discussion

In the present study, we observed that 1) RMs vaccinated with Rev-Ind Nef¯SIV two weeks prior to the onset of challenges with pathogenic SIVsmE660 were more susceptible to this heterologous virus compared to monkeys with much longer time intervals between live-virus exposure and SIVsmE660 challenges; 2) microarray analysis suggested that a generalized immune activation led to enhancement of chemokines and chemokine receptors, most notably CCL3 and CCR5, which may have increased host susceptibility to superinfection by the heterologous SIVsmE660 during acute infection with the vaccine strain, Rev-Ind Nef¯SIV; and 3) while Rev-Ind Nef¯SIV induced some level of protection, this protection was not absolute, as most vaccinated animals became superinfected after a high-dose SIVsmE660 challenge.

Although SIVsmE660 acquisition during multiple low-dose mucosal challenges has been reported to be influenced by TRIM5α [37], results from the present study and additional SIVsmE660 mucosal titration studies at the Yerkes NPRC suggest a moderate influence of this antiviral factor in acquisition at best. In addition such influence was even less prevalent with a higher dose mucosal challenge during which all animals became infected. Furthermore, the levels of steady-state viremia were also found to be influenced by TRIM5α alleles after infection with SIVsmE660 or the molecular clone SIVsmE543-3 [38,39].

Significant levels of protection have been achieved against primate lentivirus infection with live-attenuated SIV [2,3,40,41], although translation of such strategy to humans is unacceptable due to the residual pathogenic potential of standard *nef*-deleted vaccine viruses, including *nef*-deleted HIV-1 [21,22], and recombination between vaccine and challenge viruses [10]. While most live-attenuated SIV approaches have inactivated the accessory genes *nef* and/or *vpr*, our team has generated a unique series of replication-competent isolates lacking Rev-RRE transport elements. These Rev-independent SIV strains have a critical need for RNA transport elements from other sources for replication. Unlike SIVΔNef or Δ3, this Rev-Ind Nef¯SIV has been shown to be apathogenic even when neonatal RMs were inoculated [27], which prompted us to assess the efficacy of this live attenuated SIV strain as a function of the time interval between vaccination and challenge with a heterologous, pathogenic SIV.

To our surprise, exposure of RMs during the acute phase of live-attenuated Rev-Ind Nef¯SIV infection to the pathogenic SIVsmE660 given as repeated low-dose i.r. challenges significantly increased acquisition of the challenge virus. Earlier studies [41,42] had reported a need for maturation of the protective responses induced by vaccination with *nef*-deleted SIV, which was implied by the increased fraction of vaccinated RMs that were protected from wild-type SIV rechallenge with increasing time intervals between vaccination and pathogenic SIV challenge. However, our data indicate for the first time an enhancement of virus acquisition at a very early time point (~ 2 weeks) post vaccination, especially when comparing Groups 1 and 2 exposed to LASIV 15 or more months before SIVsmE660 challenge with Group 3 monkeys (Figure 2), leading to chronic replication of both the attenuated and challenge virus in the latter group. Our microarray data suggest that the mechanisms facilitating challenge virus acquisition may be innate and non-specific, a concept that is supported by the upregulation of chemokine and the chemokine receptor CCR5, which probably increased the number of susceptible target cells [43], even though this upregulation on potential target CD4^+^ T cells was not statistically significant according to the phenotypic analyses in blood.

The mechanisms of protective immunity elicited by the live-attenuated SIV vaccines remain somewhat unclear. Previous studies reported that transient depletion of CD8^+^ cells during chronic infection leads to a significant increase of transient plasma vRNA levels in animals vaccinated with live-attenuated SIV, even though detection of SIV specific CD8+ T cell responses were low except in lymphoid organs [10,29,44-46]. However, recent developments in the vaccine protection experiments suggest a role for other mechanisms in vivo such as ADCC, which is mediated by CD8αα^+^ natural killer cells [47,48]. Similar to other LASIV vaccinations, animals infected with Rev-Ind Nef¯SIV for over 1 year clearly had developed various levels of humoral and cell mediated responses, yet these response were generally modest compared in response to wild type SIV [45] [41]. Therefore, monkeys that had been inoculated 8 years ago were boosted at -15 months before heterologous virus challenge, in an attempt to enhance and or re-mobilize SIV specific responses. It is unclear whether such measures resulted in any difference in protective mechanisms, since there was no apparent difference between Group 1 and Group 2 RMs, relative to low or high dose heterologous mucosal challenge. One salient outcome of these studies though, was the realization of the limits of limit of protection afforded by LASIV in a model that used heterologous mucosal challenge. Superinfection was achieved, especially when higher inoculum exposures were performed, although some control of chronic viremia was still achieved in Groups 1 and 2 vaccinees post infection, especially for those RMs superinfected by low dose challenges (Figure 2C, F and L).

We have identified acute infection with a live attenuated vaccine strain as a cause for increased acquisition of heterologous, pathogenic challenge virus. The mechanism(s) involved upregulation of innate immunity associated genes. Acute infection with other organisms could result in similarly increased rates of lentivirus acquisition, which has been demonstrated for herpes simplex virus type-2 (HSV-2) [49-52] and parasites [53,54]. Modulation of HIV acquisition rates has been reported during *de novo* HSV-2 infection in epidemiologic studies that found a 7-fold increase in the risk of HIV acquisition [51,52]. In contrast, although chronic HSV-2 infection was also associated with increased HIV-1 acquisition, the increases were only 2-3 fold [49]. Interestingly, the STEP trial, in which the efficacy of adenovirus 5 (Ad5) vectors encoding HIV *gag, pol* and *nef* was tested in human volunteers [55], vaccine recipients with pre-existing HSV-2 infection had a 3-fold higher risk of HIV-1 acquisition compared to individuals that were seronegative for HSV-2; this increased risk was noted after adjustments for other risk factors, such as Ad5 titers and circumcision [56]. Though a clear mechanism accounting for increased susceptibility to infection remains to be identified, immunization with select Ad5 vectors induced significantly greater median percentages of activated CCR5 ^+^ CD4^+^ T cells, suggesting that these cells may have migrated to mucosal sites, thereby increasing the number of target cells available for HIV infection and enhancing HIV acquisition [57]. In a primate model of HSV-2/SHIV, upregulation of several of chemokines and cytokines was described [50]. Acute parasite infection of RMs also significantly increased host susceptibility to SHIV acquisition, as our previous studies demonstrated [53,54]. During acute *Schistosoma mansoni* infection, the SHIV dose required to achieve systemic infection after mucosal exposure was 17-fold lower in parasitized RMs compared to their parasite-free counterparts [53,54]. We have noted upregulation of several cytokines during acute infection with parasites [53,54]. The increased susceptibility to SHIV acquisition was linked to mucosal as opposed to intravenous SHIV challenges [54].

Our findings regarding live-attenuated Rev-Ind Nef¯SIV as well as examples from the literature demonstrate that inflammation or immune activation at acute stages of infection, be it with a live-attenuated vaccine strain, HSV-2, or parasites increase host susceptibility to *de novo* HIV/SHIV infection. Thus, our data highlight the need to consider enhancement of lentiviral acquisition during acute-stage infection with infectious agents-cum vaccine candidates. For vaccine trials, live-vector vaccination may require special warnings to vaccine recipients regarding increased risks of HIV acquisition during the acute phase vaccine vector replication.

## Materials and Methods

### Animals

Indian RMs were housed at the Yerkes National Primate Research Center (YNPRC, Emory University, Atlanta, GA) and handled according to the National Institutes of Health guidelines on the care and use of laboratory animals. Animal experiments were carried out in strict accordance with the recommendations in the Guide for the Care and Use of Laboratory Animals of the U.S. Public Health Services/National Institutes of Health, as well as according to the recommendations in the Weatherall report on ‘‘The Use of Non-human Primates in Research’’ (http://www.acmedsci.ac.uk/images/project/nhpdownl.pdf). The protocol was approved by the Committee on the Ethics of Animal Experiments of Emory University (IACUC ID: YER-256-2008Y; Emory University Animal Welfare Assurance Number A3180-01). The animals were housed indoors with a 12 hour light/dark cycle, in individual cages but in visual and auditory contact with other RMs, were fed monkey chow (Purina) ad libitum supplemented daily with fresh fruit. Standard enrichment was provided by the YNPRC enrichment staff including perches, rubber toys and varied treats such as peanuts and cereals. Blood was collected under ketamine or Telazol anesthesia from the femoral vein. Animals were closely monitored and observed for development of disease at least twice daily. Animals determined to be under stress or in discomfort, were administered appropriate anesthetics and/or analgesics as directed by the clinical veterinary staff. Monkeys showing signs of disease or distress or reaches IACUC endpoints, such as pain or stress that could not be alleviated using standard analgesics and/or chemotherapy were humanely euthanized with an intravenous overdose of pentobarbital sodium according to the guidelines of the American Veterinary Medical Association. Distribution of RMs into the various groups is illustrated in Figure 1; Determination of the major histocompatibility complex (MHC) or Tripartite-motif 5 alpha (TRIM5α) genotyping were done after the initiation of the study given the fact that such tools were not available at the study onset.

### Virus stocks

Virus stocks were generated in peripheral blood mononuclear cells (PBMC) collected from healthy, pathogen-free RMs and were titrated *in vitro* using CEMx174-GFP cells [58]. Rev-Ind Nef¯SIV was generated in our laboratories [25] and SIVsmE660 was obtained from the AIDS Research & Reference Reagents Program (ARRRP), National Institute of Allergy and Infectious Diseases (NIAID), National Institutes of Health (NIH), Bethesda, MD, USA. Immunizations with Rev-Ind Nef¯SIV were given by the i.v. route.

### Mucosal SIV challenges

Ketamine-anesthetized RMs received either low-dose or high-dose i.r. inoculations of SIVsmE660 in a total volume of one ml through a gastric feeding tube, which was, inserted 5 cm into the rectum with the aid of a lubricant. Virus administration (300 50% tissue culture infectious doses (TCID_50_) for each low and 990 TCID_50_ for the high-dose challenges) was followed by two ml of supplement-free RPMI-1640 medium and one ml of air. The animals’ pelvis were left elevated for 15 min before the RMs were returned to their cages.

### Plasma vRNA levels

RNA was isolated using the QiaAmp Viral RNA Mini-Kit (Qiagen, Valencia, CA), and vRNA levels were measured by quantitative RT-PCR; the assay sensitivity was 50 vRNA copies/ml [32,33].

### Differential PCR to distinguish between Rev-Ind Nef¯SIV and SIVsmE660

We used differential PCR to detect the vaccine (Rev-Ind Nef¯SIV) and challenge (SIVsmE660) viruses. SIVmac251-specific primers and probes developed by Cline et al. [33] were used to detect vRNA from both Rev-Ind Nef¯SIV (vaccine virus) and SIVsmE660 (challenge virus). Differentiation of the Rev-Ind Nef¯SIV vaccine virus from the challenge virus SIVsmE660 in the same vRNA sample was performed with primers and probes specific for SIVmac239 developed by Hofmann-Lehmann et al. [32].

The differences between these two assays reflect vRNA loads of SIVsmE660 superinfection.

### ELISPOT assays

PBMC were tested for virus-specific responses using overlapping Gag peptide pools of SIVmac239 (ARRRP) as described previously [27,59]. For long-term ELISPOT assays, cells were cultured at a final concentration of 5 × 10^6^ cells per ml in 24-well plates for 10 days with SIVmac239 overlapping Gag peptide pools at a final concentration of 2 µg/ml of each peptide. Interleukin-2 (IL-2) was added to the cultures every 3 days at a final concentration of 10 U/ml.

### MHC class I and TRIM5 α typing of macaques

All RMs were typed for MHC and TRIM5α after completion of the study since genotyping was not routinely performed at the time of initiation of the current study. However, it has since been reported that select MHC class I alleles are associated with delayed SIV disease progression [60,61] and therefore, typing for Mamu-A*001, B*008 and B*017 (Table 1) was performed to evaluate the distribution of these alleles into the various groups. TRIM5α genotypes of RMs challenged with SIVsmE660 were determined as published [38]. In brief, DNA was isolated from cryopreserved PBMC and genotyping was performed at the Genetics Core of the New England Primate Research Center.

### PBMC and rectal biopsy collections

For microarray analysis, blood was collected in sodium citrate CPT tubes and centrifuged immediately according to the manufacturer’s instructions to separate PBMC and plasma; PBMC were stored in RNAlater solution (Ambion, Austin, TX) at -80°C. Similarly, rectal pinch biopsies were collected 2 weeks before and after the administration of Rev-Ind Nef¯SIV. Rectal pinch biopsies were collected and immediately placed into RNAlater solution and stored -80°C until further processing. We allowed 2 weeks for the rectal tissues to heal before i.v. inoculation with Rev-Ind Nef¯SIV, the live attenuated vaccine strain.

### RNA extraction and microarray analysis

Total RNA from RMs PBMC and rectal biopsies was extracted using RNAeasy extraction kits (Qiagen, Valencia, CA). cDNA labeling, hybridization, staining and scanning were performed according to the manufacturer’s instructions (Affymetrix, Santa Clara, CA).

### Microarray data analysis

Array quality was assessed using the R/Bioconductor package [62]. Affymetrix CEL files were processed and normalized using the robust multiarray average (RMA) algorithm [63]. Principal component analysis (PCA) was performed on the normalized expression values to identify phenotypically similar groups by visualizing the top three principal components. To identify genes correlating with the phenotypic groups, the data were fitted to a linear model using the limma package [64] and tested for differential gene expression. Results were adjusted for multiple testing using the Benjamini and Hochberg (BH) method [65], and significance was determined using a false-discovery-rate cutoff of <5% and a log fold-change cutoff of >1. Expression values of significant probe sets were loaded into the MultiExperiment Viewer (MeV) [66] and analyzed using average-linkage hierarchical clustering with a Pearson correlation coefficient distance metric. Gene interaction network analysis and visualization were performed on significant probe sets using the Ingenuity pathway analysis (IPA) software package. Microarray data was deposited into the gene expression omnibus database (GSE49663).

### Statistical analyses

The log-rank test was used to compare the time-to-peak viremia distributions between the groups. The Wilcoxon-rank sum test was used to compare peak viremia between groups. The Montel-cox test was used to compare difference between TRIM5α and viral acquisition. All *P*-values reported are based on two-sided tests.

## Supporting Information

Figure S1 **Analysis of CD4 T cell subsets in Group 7 (n=3) animals, inoculated with Rev-Ind Nef¯SIV.** Data shown are comparison of samples collected before and 2 weeks post-infection A) Absolute CD4^+^ T cells B) CD4^+^ CD45RA^-^CCR5^+^ C) CD4^+^ CD45RA^-^CCR7^+^ and D) Central Memory Cells (CD4^+^ CD28^+^ CD95^+^).
(TIF)
