# Supplementary figures and images for: Live Attenuated Rev-Independent Nef¯SIV Enhances Acquisition of Heterologous SIVsmE660 in Acutely Vaccinated Rhesus Macaques
Source: PLoS One. 2013 Sep 30;8(9):e75556. doi: 10.1371/journal.pone.0075556 (PMC3787041; doi:10.1371/journal.pone.0075556)

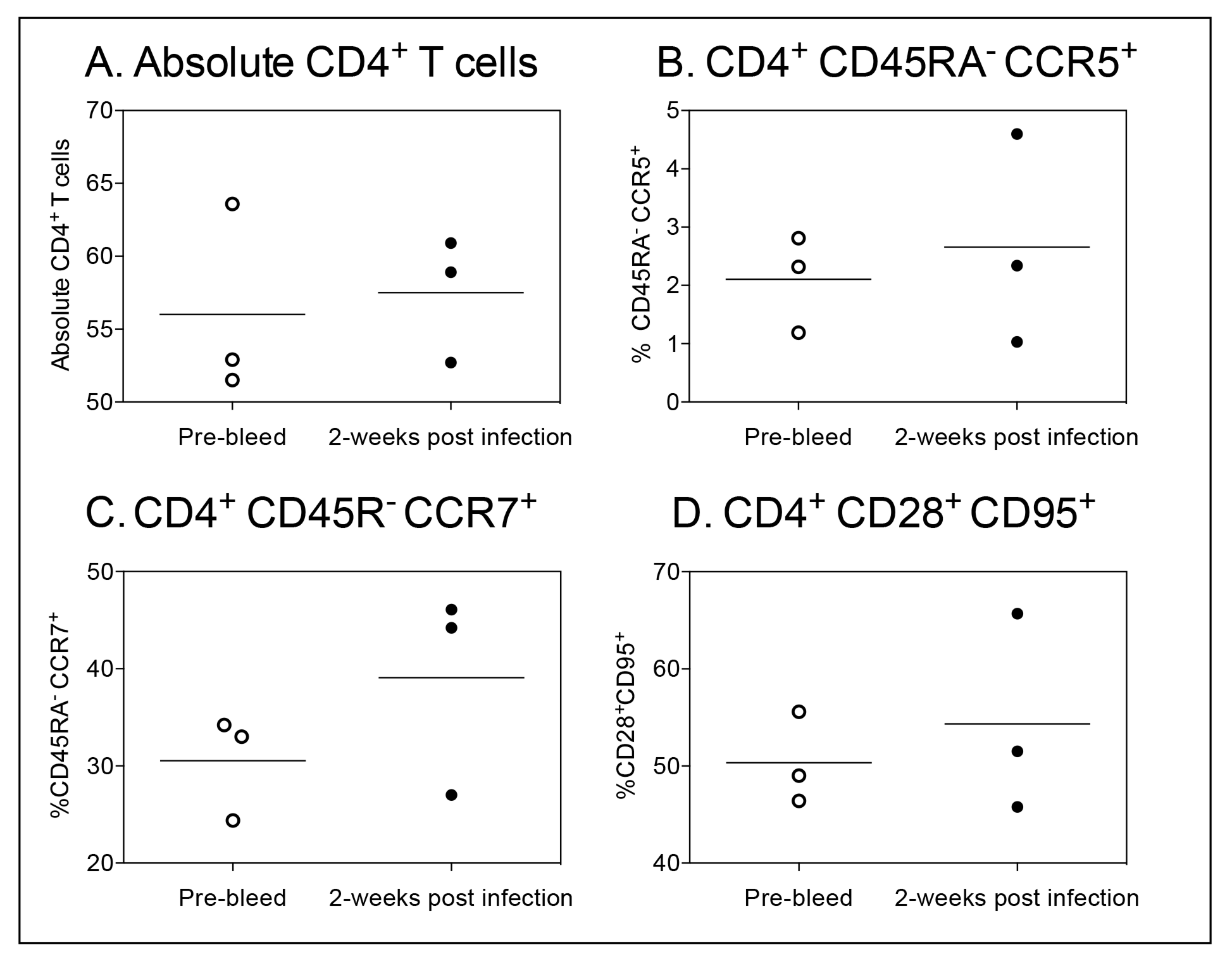

Supplement: Figure S1 — Analysis of CD4 T cell subsets in Group 7 (n=3) animals, inoculated with Rev-Ind Nef¯SIV. Data shown are comparison of samples collected before and 2 weeks post-infection A) Absolute CD4+ T cells B) CD4+ CD45RA-CCR5+ C) CD4+ CD45RA-CCR7+ and D) Central Memory Cells (CD4+ CD28+ CD95+). (TIF) [file pone.0075556.s001.tif]
